# Long noncoding RNA LINC02418 regulates MELK expression by acting as a ceRNA and may serve as a diagnostic marker for colorectal cancer

**DOI:** 10.1038/s41419-019-1804-x

**Published:** 2019-07-29

**Authors:** Yinghui Zhao, Tiantian Du, Lutao Du, Peilong Li, Juan Li, Weili Duan, Yunshan Wang, Chuanxin Wang

**Affiliations:** 1 Department of Clinical Laboratory, The Second Hospital of Shandong University, 247 Beiyuan Street, Jinan, 250033 Shandong China; 2 Tumor Marker Detection Engineering Technology Research Center of Shandong Province, Jinan, Shandong China; 3 Tumor Marker Detection Engineering Laboratory of Shandong Province, Jinan, Shandong China; 4 The Laboratory Clinical Medical Research Center of Shandong Province, Jinan, Shandong China

**Keywords:** Tumour biomarkers, Diagnostic markers

## Abstract

Some types of long noncoding RNAs (lncRNAs) are aberrantly expressed in human diseases, including cancer. However, the overall biological roles and clinical significances of most lncRNAs in colorectal cancer (CRC) are not fully understood. First, The Cancer Genome Atlas (TCGA) was analyzed to identify differentially expressed lncRNAs between CRC tissues and noncancerous tissues. We identified that LINC02418 was highly expressed in CRC tissues and cell lines. Next, we evaluated the effect of LINC02418 on CRC tumorigenesis and its regulatory functions of absorbing microRNA and indirectly stimulating protein expression by acting as a ceRNA. Mechanistically, LINC02418 acted as a ceRNA to upregulate MELK expression by absorbing miR-1273g-3p. In addition, the diagnostic performance of cell-free LINC02418 and exosomal LINC02418 were both evaluated by the receiver operating characteristic curve and the area under the curve (AUC). Exosomal LINC02418 could distinguish the patients with CRC from the healthy controls (AUC = 0.8978, 95% confidence interval = 0.8644–0.9351) better than cell-free LINC02418 (AUC = 0.6784, 95% confidence interval = 0.6116–0.7452). Collectively, we determined that LINC02418 was significantly overexpressed in CRC and that the LINC02418–miR-1273g-3p–MELK axis played a critical role in CRC tumorigenesis. Finally, exosomal LINC02418 is a promising, novel biomarker that can be used for the clinical diagnosis of CRC.

## Introduction

Colorectal cancer (CRC) is one of the most common malignant tumors, and its mortality ranks third among male and female malignancies worldwide^1^. Despite long-term studies, the mechanism of CRC tumorigenesis has not been fully elucidated, and effective diagnostic methods have not been fully identified until recently. When diagnosed with CRC, most patients are already in the middle or late stage and lose the opportunity for timely and standard treatment^2^. Therefore, early detection and diagnosis is the key to the treatment of CRC.

At present, the methods available for the early diagnosis of CRC are very limited owing to unreliable detection and poor compliance, such as colonoscopies, fecal occult blood tests, and imaging examinations^3,4^. Easily accessible biomarkers, such as serum biomarkers, are important tools for optimizing patient diagnosis in patients with CRC^5^. The classical serum biomarkers for CRC clinical diagnosis include carcinoembryonic antigen (CEA) and carbohydrate antigen 19–9. However, these biomarkers lead to some false-positive and false-negative results in CRC diagnosis due to the low sensitivities and specificities^6,7^. Therefore, novel noninvasive molecular diagnostic biomarkers with high sensitivities and specificities are still urgently needed.

Increasing evidence has indicated that circulating cell-free long noncoding RNAs (lncRNAs) in the serum or plasma are promising candidate biomarkers for detecting, monitoring, and predicting malignant tumors, such as prostate cancer^8^, breast cancer^9^, gastric cancer^10^, and CRC^11^. LncRNAs are a class of transcripts with lengths longer than 200 nucleotides that control a wide range of physiological and pathological processes, such as transcription, translation, cell cycle regulation, and stem cell pluripotency^12–14^. The clinical application, biological function, and underlying mechanisms in the malignancy of several important lncRNAs have been characterized in recent years. For example, the lncRNA TSLNC8 is a promising prognostic predictor for patients with hepatocellular carcinoma^15^. The lncRNA PCA3 has been routinely used in the clinic for the diagnosis of prostate cancer^16^^.^ Our previous studies have shown that the expression of several lncRNAs was significantly increased in tumor patients compared with healthy individuals^17,18^, which provide a new method for the early diagnosis of tumors.

Although a growing number of lncRNAs have been annotated, the overall biological roles and clinical significance of most lncRNAs in CRC are still unclear. In this study, we identified a novel lncRNA associated with tumorigenesis, LINC02418, was highly expressed in CRC cell lines and tissues. However, its contribution to CRC tumorigenesis and whether LINC02418 in serum can effectively diagnose CRC still remain uncertain. Here, our research focused on the revelation of the regulatory mechanism of LINC02418 in the development of CRC and may provide a novel diagnostic biomarker for CRC patients.

## Results

### LINC02418 is significantly upregulated in CRC

**Fig. 1:**
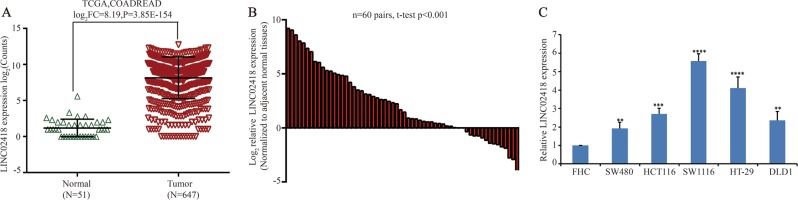
LINC02418 was upregulated in CRC. **a** Relative expression of LINC02418 in CRC compared with normal tissue was analyzed by using TCGA database. **b** LINC02418 expression was measured in 60 pairs of CRC and corresponding adjacent non-tumor tissues using qPCR. **c** LINC02418 expression was measured in normal colon epithelial cell line (FHC) and established CRC cell lines (DLD-1, SW480, HT29, HCT116, SW1116, LOVO) using qPCR. The data are presented as the means + SD from three biological replicates. **p < 0.01, ***p < 0.001, ****p < 0.0001

To investigate the differential gene expression in CRC, we first analyzed the RNA sequencing data of a colorectal carcinoma (COADREAD) cohort study containing 51 normal and 647 CRC tissues from The Cancer Genome Atlas (TCGA) database. The result showed that LINC02418 expression was significantly higher in CRC tumors than in non-tumor tissues (Fig. 1a). LINC02418 is located on chromosome 8 (GRCh38.p7, 130033454-130042342). Online bioinformatics analysis revealed that LINC02418 had no coding capability (http://cpc.cbi.pku.edu.cn/programs/run_cpc.jsp). Upon evaluating 60 pairs of clinical colorectal tissues by quantitative PCR (qPCR), a significantly higher level of LINC02418 was detected in CRC tumors compared with matched adjacent normal tissues (Fig. 1b). The correlation between LINC02418 expression in tissues and the clinic-pathological characteristics of the CRC patients was analyzed (Table S1). There was no association between the expressions of LINC02418 and patient age, sex, tumor size, lymph node metastasis, or TNM stage. A higher expression of LINC02418 was also observed in established CRC cell lines (DLD-1, SW480, HT29, HCT116, and SW1116) compared with the immortalized normal colon epithelial cell line FHC (Fig. 1c). We chose the SW1116 and HT29 cell lines for further studies because they exhibited the highest expression levels compared with FHC. These results demonstrated that LINC02418 was highly expressed in CRC.

### LINC02418 is required for the efficient proliferation of CRC cells

**Fig. 2:**
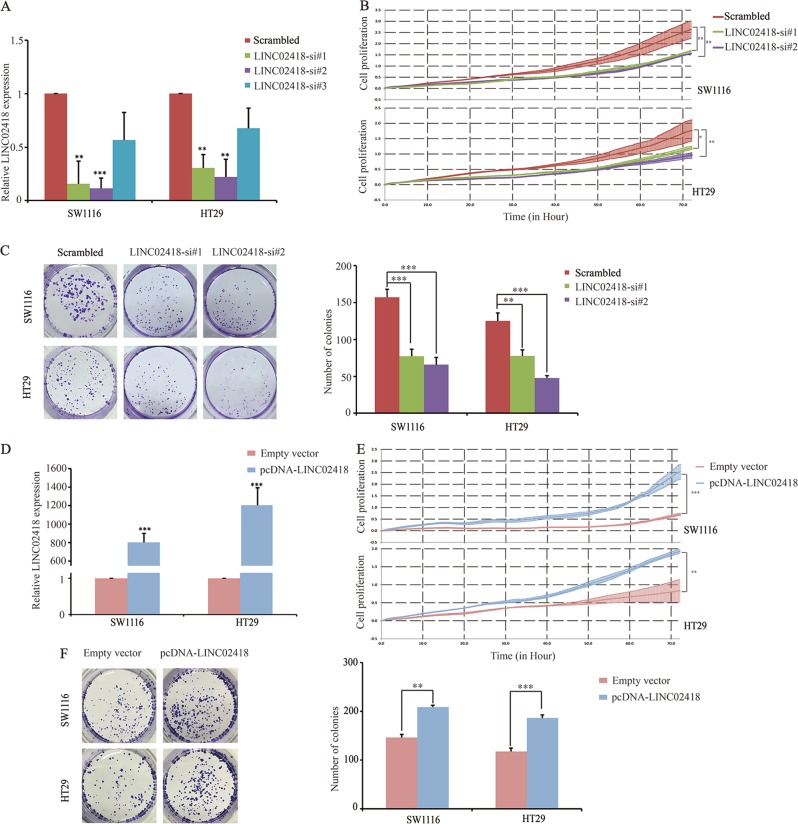
Effects of LINC02418 on CRC cells proliferation. **a**, **d** qPCR analysis of LINC02418 expression in control, si-LINC02418 1#, si-LINC02418 2#, si-LINC02418 3#, and pcDNA-LINC02418-treated CRC cells. **b**, **e** xCELLigence system was used to monitor the cell growth dynamics of si-LINC02418-transfected or pcDNA-LINC02418-transfected CRC cells. **c**, **f** Colony formation assay was performed to determine the proliferation of si-LINC02418-transfected or pcDNA-LINC02418-transfected CRC cells. All experiments were repeated at least three times, and representative data are shown. Data are means ± SEM. **p < 0.01, ***p < 0.001

To investigate the biological functions of LINC02418 in the tumorigenesis and development of CRC, three specific siRNAs were used to knockdown LINC02418 expression in two CRC cell lines, SW1116, and HT29. The qPCR analyses showed that of the three siRNAs, si-LINC02418 1# and 2# exhibited better silencing efficiency (Fig. 2a). The growth curves generated from the cell growth dynamics (monitored using the xCELLigence system) continuously showed that LINC02418 knockdown significantly inhibited CRC cell proliferation (Fig. 2b). Similarly, a colony formation assay also confirmed that LINC02418 downregulation markedly reduced the colony formation number of CRC cells (Fig. 2c). In contrast, an overexpression plasmid (pcDNA3.1-LINC02418) was transfected to increase the ectopic expression of LINC02418 (Fig. 2d). The results of the RTCA xCELLigence experiments and the colony formation assays showed that LINC02418 overexpression promoted CRC cell growth ability and colony-forming capacity (Fig. 2e, f). The above findings indicate that LINC02418 behaves as an oncogene that is required for the efficient proliferation of CRC cells.

### LINC02418 silencing inhibits CRC cell proliferation by inducing cell cycle arrest and promoting apoptosis

**Fig. 3:**
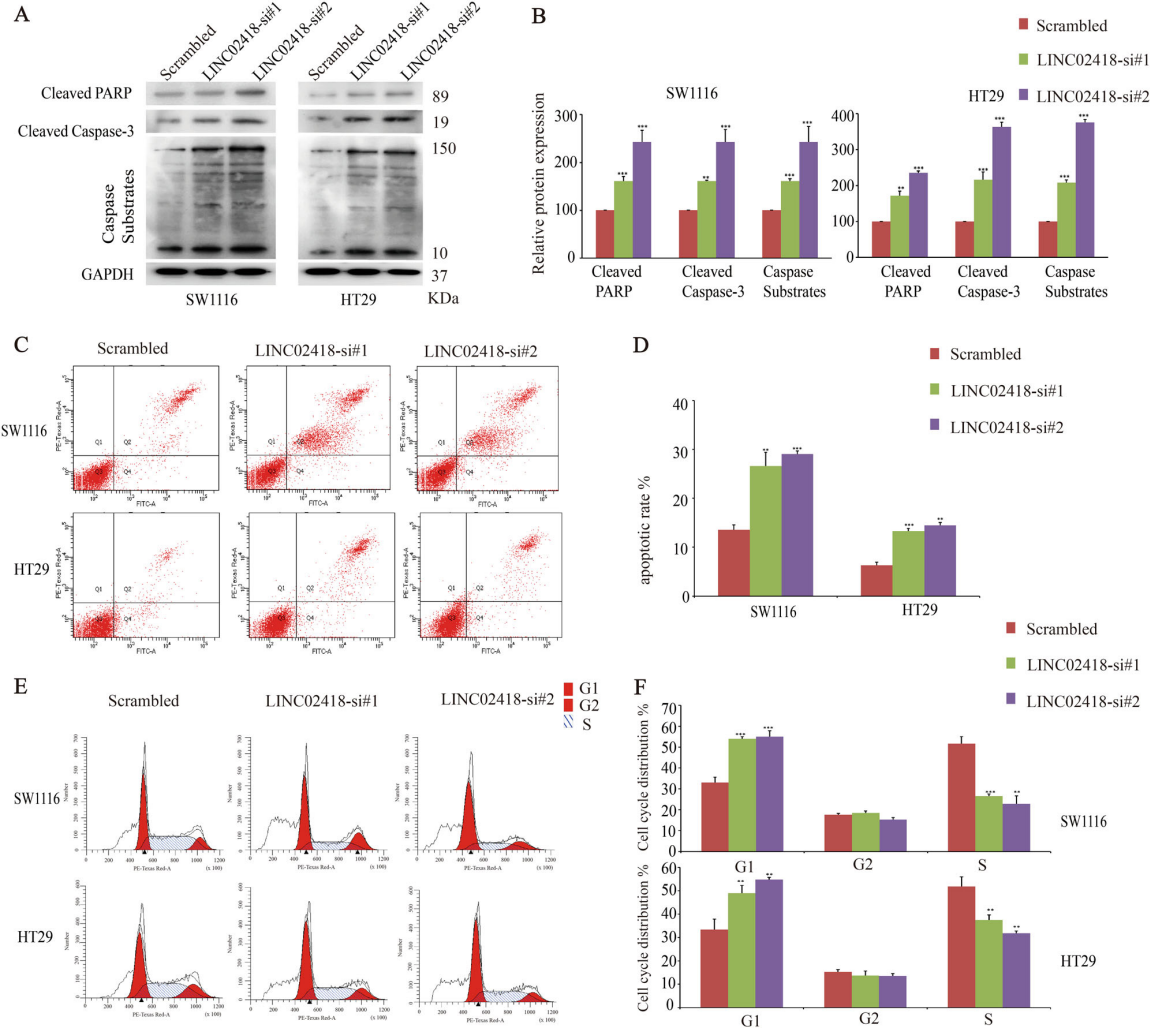
Effects of LINC02418 on CRC cells apoptosis and cell cycle. **a**, **b** Representative western blots of apoptosis-related proteins after negative control siRNA, si-LINC02418 1#, or si-LINC02418 2# transfection in CRC cells. GAPGH protein was used as an internal control, and the data from the “scrambled” are arbitrarily set to 100% (right panel). **c**, **d** Flow cytometry was used to detect the apoptotic rates of cells (LR + UR). LR, early apoptotic cells; UR, terminal apoptotic cells. **e**, **f** The cycle distributions of si-LINC02418-transfected CRC cells were detected by flow cytometry. All experiments were repeated at least three times, and representative data are shown. Data are means ± SEM. **p < 0.01, ***p < 0.001

To determine the molecular mechanism by which LINC02418 suppresses cell proliferation, we investigated the effect of LINC02418 knockdown on apoptosis and cell cycle distribution. First, LINC02418 silencing can induce a significant elevation in apoptosis-related protein levels, including cleaved Caspase-3, cleaved PARP and caspase substrates, as was shown in the SW1116 and HT29 cell lines by western blots (Fig. 3a, b). Consistently, an apoptosis assay by flow cytometry showed that LINC02418 knockdown induced significant increases in both the early and late phase apoptotic cells compared with the scrambled negative control group in the SW1116 and HT29 cells (Fig. 3c, d). Meanwhile, to detect whether LINC02418 controls the cell cycle, flow cytometry was applied to investigate the percentage of cells in the G1, S, and G2 phases (Fig. 3e, f). The results revealed that the silencing of LINC02418 obviously increased the number of cells in the G1 phase and significantly decreased the number of cells in the S phase in the SW1116 and HT29 cells concomitantly. These data indicate that the inhibition of CRC cell proliferation by LINC02418 silencing could be attributed to affecting both apoptosis and the cell cycle.

### LINC02418 regulates MELK expression by functioning as a ceRNA and sequesters miR-1273g-3p

**Fig. 4:**
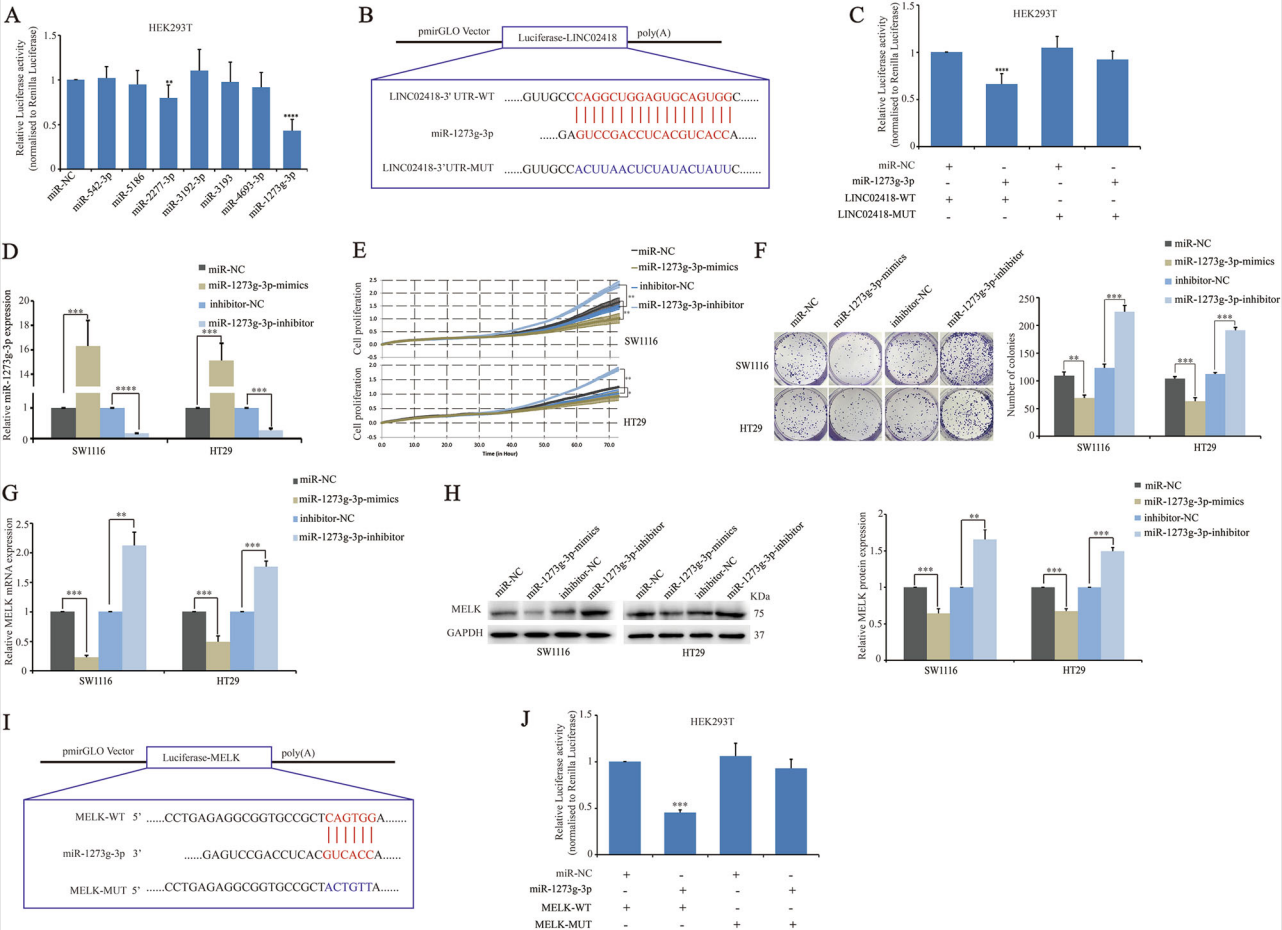
miR-1273g-3p directly targets MELK and LINC02418. **a** The pmirGLO-LINC02418 luciferase reporter plasmid was co-transfected into HEK293T cells with miRNA negative control and seven various miRNA mimics. **b** Schematic representation of WT- and Mut-LINC02418 sequences. Blue fonts represented the mutant bases. **c** The luciferase reporter plasmid containing WT- and Mut-LINC02418 was co-transfected into HEK293T cells with miR-1273g-3p in parallel with an empty plasmid vector. **d** MiR-1273g-3p expression was detected in CRC cells by qPCR after transfection of miR-1273g-3p mimics, miR-1273g-3p inhibitor, or control. **e** xCELLigence system was used to monitor the cell growth dynamics when loss and gain of miR-1273g-3p. **f** Colony formation assay was performed to determine the proliferation of when loss and gain of miR-1273g-3p. **g**, **h** Relative mRNA and protein levels of MELK in CRC cells transfected with miR-1273g-3p mimics, miR-1273g-3p inhibitor, or control. **i** Schematic representation of WT- and Mut-MELK sequences. Blue fonts represented the mutant bases. **j** The luciferase reporter plasmid containing WT- and Mut-MELK was co-transfected into HEK293T cells with miR-1273g-3p in parallel with an empty plasmid vector. **p < 0.01, ***p < 0.001, ****p < 0.0001

It is known that lncRNAs can function as competing endogenous RNAs (ceRNAs) to indirectly stimulate protein expression by competing for shared microRNAs. We predicted and screened the candidate microRNA targets of LINC02418 using the bioinformatics tool miRanda. MiR-1273g-3p, miR-542-3p, miR-5186, miR-2277-3p, miR-3192-3p, miR-3193, and miR-4693-3p were predicted as the most likely microRNAs to complement LINC02418 (Supplementary Fig. S1). To determine whether any of these candidate miRNAs is regulated by LINC02418, the cDNA fragments of LINC02418 were cloned into the pmirGLO-Basic luciferase reporter vector and were co-transfected into HEK293T cells along with the seven miRNAs and the control miRNA mimics. Among these candidate miRNAs, only miR-2277-3p and miR-1273g-3p could suppress the LINC02418-driven luciferase activity, and the suppression ability of miR-1273g-3p was stronger (Fig. 4a). Hence, we chose miR-1273g-3p as a candidate for further investigation and constructed a reporter vector in which the putative miR-1273g-3p-binding site in the LINC02418 sequence was mutated by base mutations (Fig. 4b) and co-transfected the vector into HEK293T cells with control miRNA and miR-1273g-3p mimics. As expected, it was observed that miR-1273g-3p decreased the luciferase activity in the wild-type vector, instead of that in the mutant type (Fig. 4c). To determine whether miR-1273g-3p has a suppressor role in the tumorigenesis and development of CRC we transfected SW1116 and HT29 cells with miR-1273g-3p mimic or inhibitor (Fig. 4d). Then, we performed RTCA xCELLigence experiments and the colony formation assays and found that cell growth ability and colony-forming capacity were reduced by overexpression of miR-1273g-3p and increased by inhibition of miR-1273g-3p expression (Fig. 4e, f).

Using TargetScan software, we identified that MELK may be a potential target of miR-1273g-3p. To determine whether MELK is regulated by miR-1273g-3p in CRC cells, we measured MELK mRNA and protein levels when miR-1273g-3p was overexpressed or inhibited in SW1116 and HT29 cells. The mRNA and protein levels of MELK were significantly decreased or increased by miR-1273g-3p overexpression or inhibition, respectively (Fig. 4g, h). Then, the luciferase reporter assays were performed by co-transfected the wild-type sequence of MELK or mutant constructs containing a mutation in the miR-1273g-3p-binding sites (Fig. 4i), with control miRNA and miR-1273g-3p mimics. The results showed that wild-type of luciferase expression was significantly reduced by miR-1273g-3p mimics compared with that in the mutant type (Fig. 4j), which provided evidence that miR-1273g-3p can directly target MELK.

**Fig. 5:**
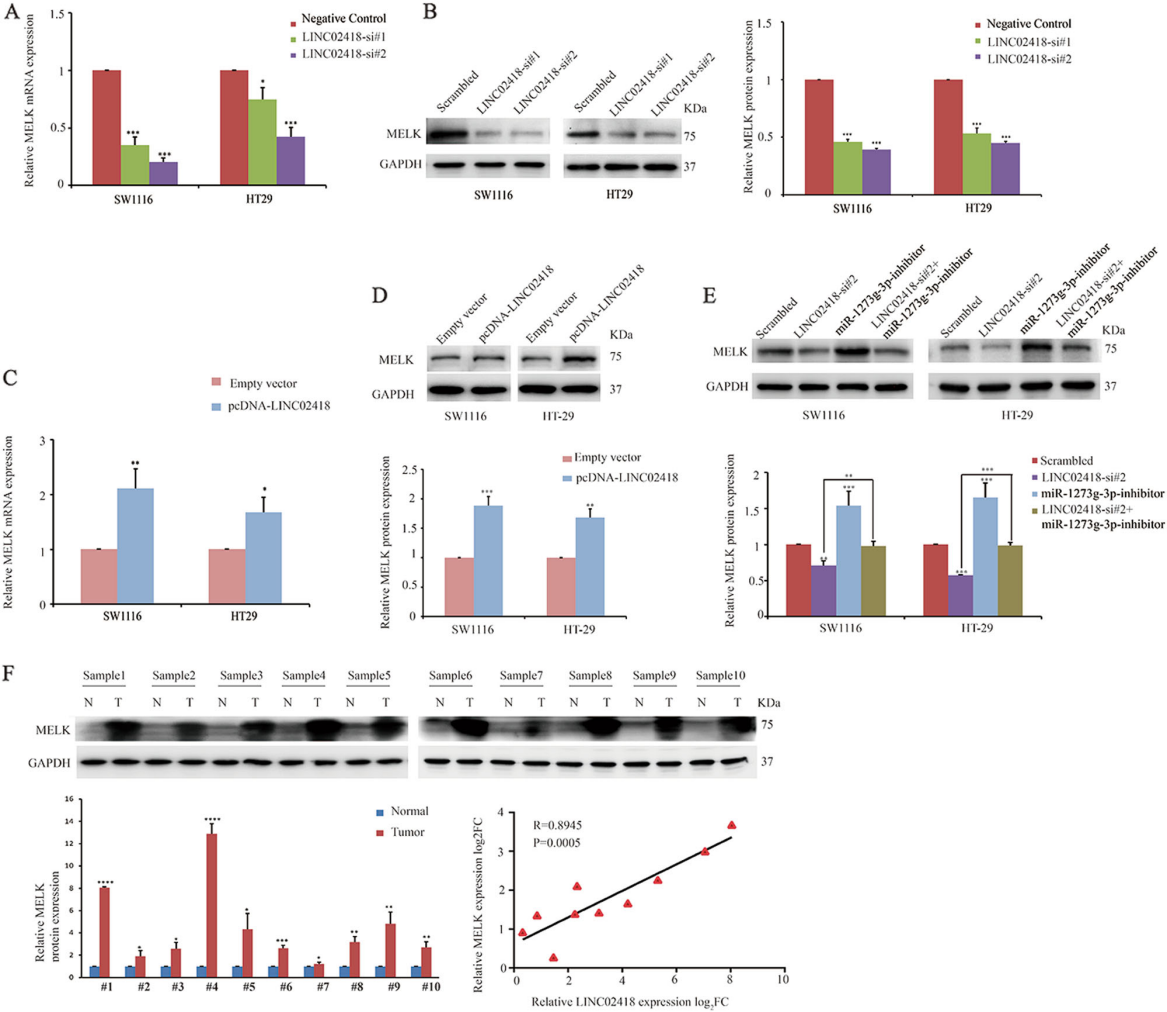
LINC02418 regulates MELK expression via functioning as a ceRNA and sponging miR-1273g-3p. **a**, **b** Relative mRNA and protein levels of MELK in CRC cells transfected with si-LINC02418 1#, si-LINC02418 2#, or negative control. **c**, **d** Relative mRNA and protein levels of MELK in CRC cells transfected with pcDNA-LINC02418 and empty vectors. **e** Relative protein levels of MELK in CRC cells following knockdown of LINC02418 and/or inhibition of miR-1273g-3p. **f** Relative protein levels of MELK in 10 pairs of CRC and adjacent normal tissues (upper), measured using the ImageJ software and normalized to the expression of GAPDH (lower left). Relationship between MELK and LINC02418 was measured (lower right). All experiments were repeated at least three times, and representative data are shown. Data are means ± SEM. **p < 0.01, ***p < 0.001, ****p < 0.0001

To determine the ceRNA network between LINC02418 and MELK in CRC, we transfected CRC cells with si-LINC02418 1# and 2# and the results showed that the knockdown of LINC02418 also significantly reduced the MELK mRNA and protein levels in SW1116 and HT29 cells (Fig. 5a, b). In contrast, the mRNA and protein levels of MELK were significantly increased by LINC02418 overexpression (Fig. 5c, d). Next, we want to examine whether miR-1273g-3p has a role in the relationship between LINC02418 and MELK, SW1116, and HT29 cells were co-transfected with si-LINC02418 2# and the miR-1273g-3p inhibitor. Indeed, the suppression of MELK protein levels induced by si-LINC02418 2# was effectively reversed by the miR-1273g-3p inhibitor (Fig. 5e). A positive correlation was also identified for the fold change in 10 pairs of CRC tissues between the LINC02418 mRNA level and MELK expression (*R* = 0.8945, *p* = 0.0005), which suggested that MELK expression might be promoted by LINC02418 (Fig. 5f). Collectively, these data suggest a potential positive correlation between LINC02418 and MELK.

### LINC02418 regulates MELK to promote CRC tumorigenesis

**Fig. 6:**
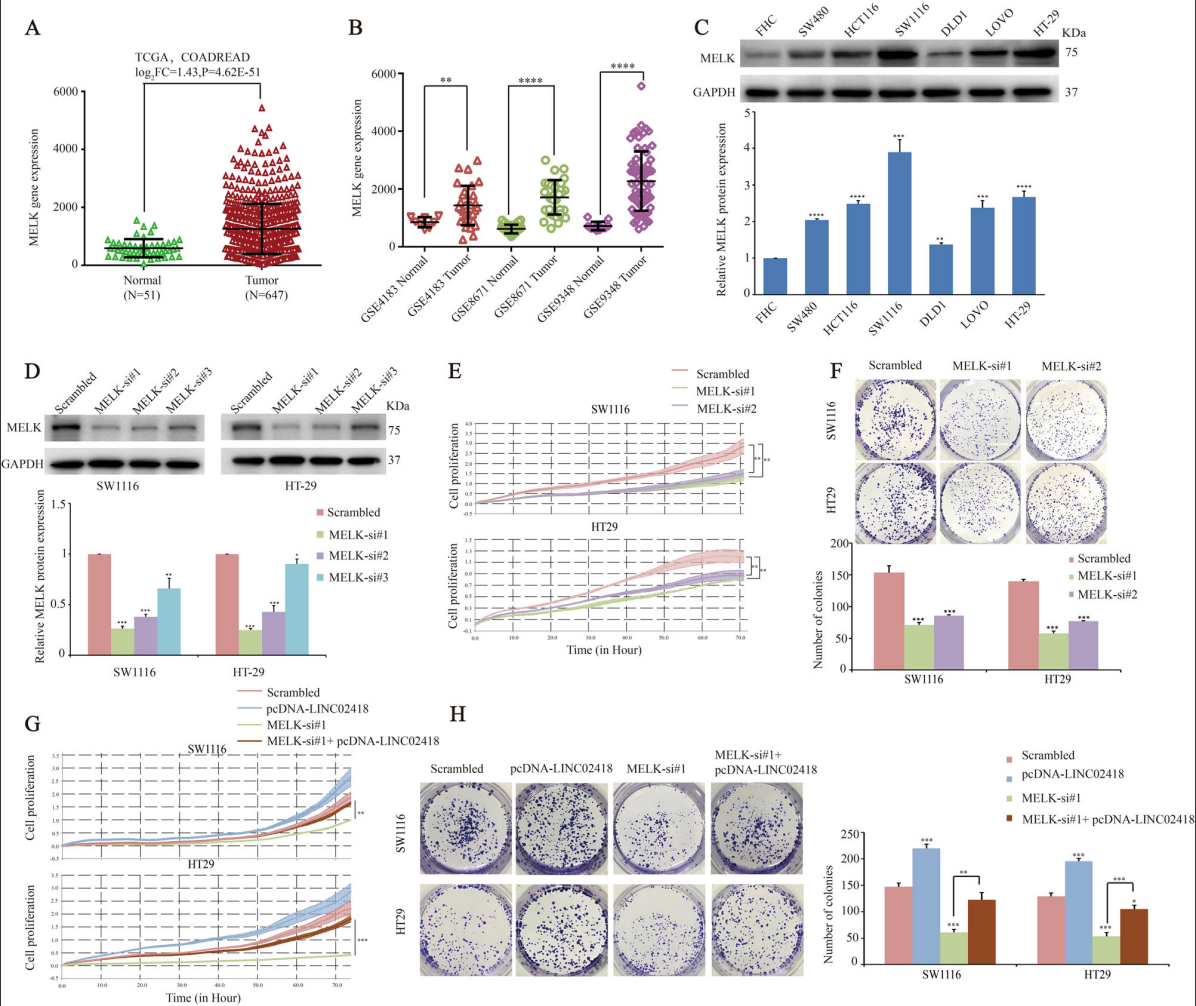
LINC02418 regulated MELK to promote CRC tumorigenesis. **a**, **b** Relative expression of MELK in CRC compared with normal tissue was analyzed by using TCGA and GEO datasets, including GSE4183, GSE8671, GSE9348. **c** Relative protein levels of MELK was measured in normal colon epithelial cell line (FHC) and established CRC cell lines (DLD-1, SW480, HT29, HCT116, SW1116, LOVO) by western blotting. **d** The MELK protein levels were determined by western blotting in MELK knockdown CRC cells. **e** xCELLigence system was used to monitor the cell growth dynamics of si-MELK-transfected CRC cells. **f** Colony formation assay was performed to determine the proliferation of si-MELK-transfected CRC cells. **g**, **h** CRC cells Growth curves and colony formation ability of CRC cell after co-transfection with negative control siRNA, si-MELK, or/and overexpression plasmid (pcDNA3.1-LINC02418) were determined by xCELLigence system and colony formation assays. All experiments were repeated at least three times, and representative data are shown. Data are means ± SEM. **p < 0.01, ***p < 0.001, ****p < 0.0001

We then asked what the biological function of MELK is in CRC cells. After analyzing the TCGA RNA sequencing data and the CRC mRNA microarray profiles from GEO datasets, we found that MELK was upregulated in CRC tissues compared with normal tissues (Fig. 6a, b). Similarly, a higher expression of MELK was also observed in established CRC cell lines (DLD-1, SW480, HT29, HCT116, SW1116, and LOVO) compared with the immortalized colon epithelial cell line FHC (Fig. 6c). Then, SW1116 and HT29 cells were transfected with MELK siRNA to knockdown MELK expression, which was confirmed by western blotting (Fig. 6d). Functionally, the results of the RTCA xCELLigence experiments showed that the knockdown of MELK expression significantly reduced the cell growth viability, and colony formation assays showed similar results (Fig. 6e, f).

To determine whether LINC02418 regulates MELK to promote CRC tumorigenesis, we examined cells co-transfected with si-MELK and the overexpression plasmid (pcDNA3.1-LINC02418). The results of the RTCA xCELLigence experiments and colony formation assays showed that the inhibition of cell growth viability caused by MELK siRNA could be partly rescued by LINC02418 overexpression (Fig. 6g, h).

### The expression of cell-free LINC02418 in the serum of CRC patients

First, the aberrant expression of cell-free LINC02418 in the serum was identified in 30 CRC patients and 20 healthy controls. We found that the expression of free circulating LINC02418 was much higher in the serum of CRC patients compared with that in healthy individuals (Supplementary Fig. S2a). Next, to validate the performance of LINC02418 in the serum for CRC diagnosis, we evaluated the expression levels of cell-free LINC02418 in another independent validation set (125 CRC patients and 125 healthy controls). Similarly, the expression of cell-free LINC02418 was clearly upregulated in CRC (Supplementary Fig. S2b). In addition, the receiver operating characteristic curve (ROC) and the area under the curve (AUC) were verified in the validation set. The AUC of the cell-free LINC02418 for CRC detection was 0.6784 (95% confidence interval = 0.6116–0.7452) (Supplementary Fig. S2c).

### Exosomal LINC02418 may serve as a diagnostic marker in CRC

The expression signatures of exosomal lncRNAs have been proposed as potential noninvasive biomarkers for cancer detection. In this study, the diagnostic performance of serum exosomal LINC02418 was evaluated in validation set further.

**Fig. 7:**
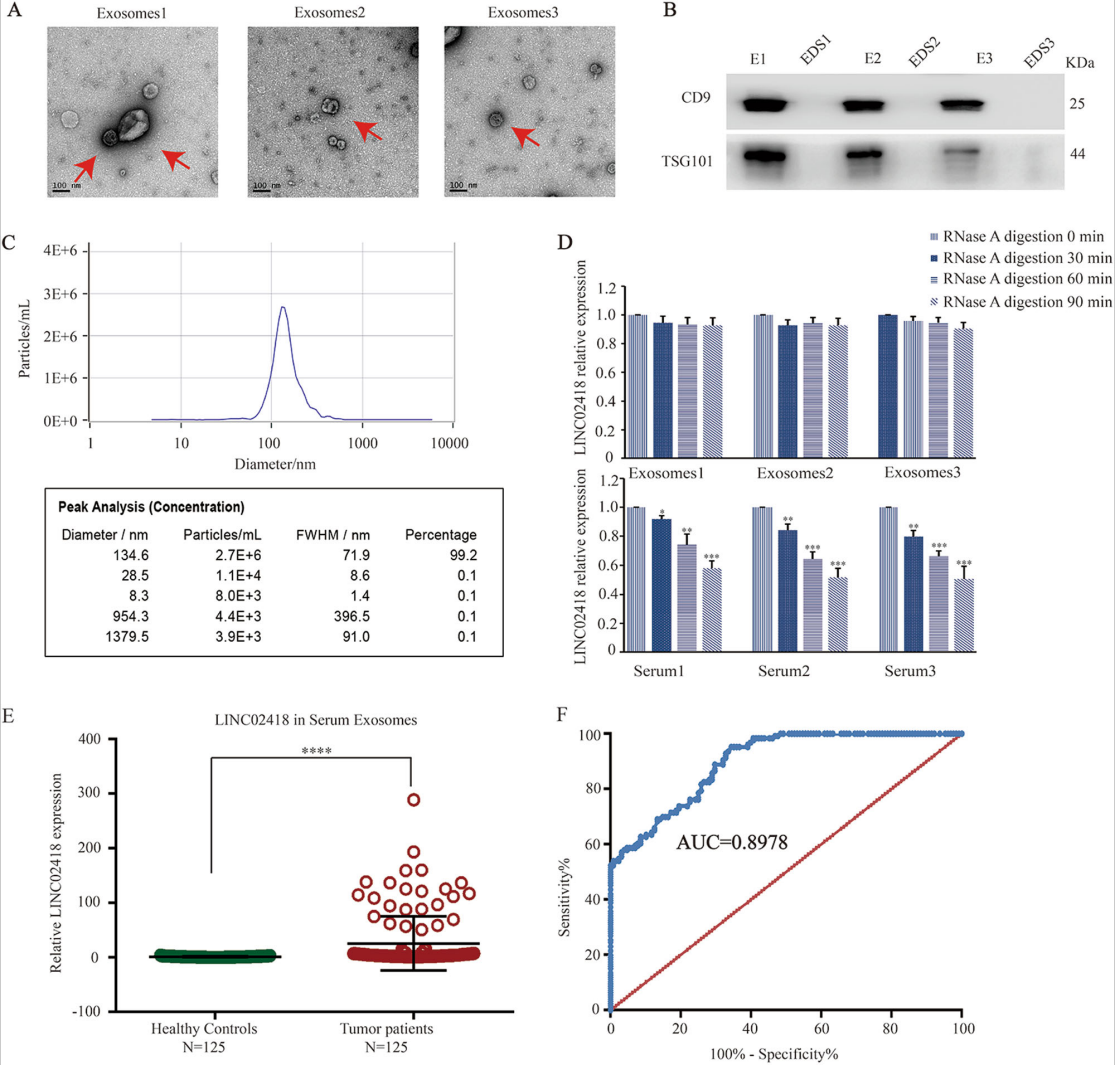
Exosomal LINC02418 may serve as a diagnostic marker in CRC. **a** Representative TEM images of serum exosomes as indicated by the arrows. Scale bar, 100 nm. **b** Western blotting analysis of TSG101 and CD9 in exosomes and exosome-depleted supernatants (EDS). **c** NTA of the size distribution and number of exosomes. **d** qPCR analysis of LINC02418 in the exosomes or in serum treated with RNase A for 0, 30, 60, and 90 min, respectively (2 mg/mL). The experiment was repeated three times and the data are means ± SEM. **e** RNA expression of exosomal LINC02418 was measured in the serum of healthy controls (*n* = 125) and the serum of CRC patients (*n* = 125) using qPCR. **f** ROC curve for serum exosomal LINC02418 for the discrimination of patients with CRC from normal healthy individuals. **p < 0.01, ***p < 0.001, ****p < 0.0001

Serum exosomes were characterized by transmission electron microscopy, western blotting, and nanoparticle tracking analysis (NTA) to ensure that the isolated exosomes from the serum were intact. These vesicles showed typical cup-shaped, round morphologies with diameters of 60–150 nm by TEM (Fig. 7a). Higher concentrations of the exosome protein markers CD9 and TSG101 were observed compared with those in exosome-depleted supernatants (Fig. 7b). NTA showed that the majority of the serum exosomes was mainly 134.6 nm in diameter (Fig. 7c). These results suggested that the exosomes were successfully isolated from the serum. Next, we investigated the stability of exosomal LINC02418. Two groups of serum samples were incubated with RNase A for 0, 30, 60, and 90 min, and then cell-free LINC02418 and exosomal LINC02418 expressions was measured respectively. Interestingly, the expression levels of the LINC02418 in exosomes remained unchanged upon RNase A treatment, while the serum cell-free LINC02418 group was obviously degraded by RNase A (Fig. 7d). Taken together, these results indicate that the LINC02418 encapsulated in the exosomes have better stability under the protection of the membrane, which is an essential prerequisite for good biomarkers.

To validate the diagnostic performance of exosomal LINC02418 in serum, the expression was measured in the same validation cohort (Fig. 7e). Moreover, the ROC curve was performed according to the expression of exosomal LINC02418 in 125 CRC patients and 125 healthy individuals (Fig. 7f). As expected, the AUC of exosomal LINC02418 for CRC detection was 0.8978 (95% confidence interval = 0.8644–0.9351), which was significantly higher than that of cell-free LINC02418 (AUC = 0.6784, 95% confidence interval = 0.6116–0.7452). The best cutoff value of serum exosomal LINC02418 for predicting CRC was 2.959 (fold change in CRC compared with healthy individuals), with a sensitivity of 95.2% and a specificity of 66.4%, which suggested that exosomal LINC02418 is a promising serum biomarker for the diagnosis of CRC. Moreover, the correlation between exosomal LINC02418 expression levels and the clinic-pathological characteristics of the CRC patients in the validation set was analyzed (Table S2). The association between the expressions of exosomal LINC02418 and clinic-pathological characteristics was not observed.

## Discussion

Here, we screened TGCA database and identified a novel CRC-associated lncRNA, LINC02418, which was significantly upregulated in CRC tissues and cell lines. Related molecular biology experiments demonstrated that the knockdown of LINC02418 inhibited cell proliferation and induced cell apoptosis, whereas the overexpression of LINC02418 promoted cell proliferation. These findings indicated that LINC02418 had a pro-malignant role in CRC tumorigenesis.

A novel regulatory mechanism has been confirmed that lncRNAs can act as endogenous molecular sponges to compete for miRNAs by binding to their shared binding sites on protein-coding mRNAs^19^. For example, the lncRNA-MEG3 functions as a ceRNA of miR-181 to regulate gastric cancer cell proliferation^20^. Another example is the lncRNA H19, which promotes glioma cell invasion and the epithelial-mesenchymal transition by acting as a ceRNA of miR-675^21^. We identified several miRNAs that may interact with LINC02418 by bioinformatics analyses. Among these miRNAs, we chose miR-1273g-3p for further studies since the luciferase activity assay confirmed the direct binding ability of the predicted miR-1273g-3p-binding site on LINC02418. MiR-1273g-3p is downregulated in various types of human cancers and functions as a tumor suppressor^22,23^.

Generally, lncRNAs exert their function by acting as ceRNAs dependent on the derepression of their miRNA targets. Hence, miRNA targets are an important part of the ceRNA network^24^. Several protein-coding genes have been identified as direct targets of miR-1273g-3p, which is of great importance in carcinogenesis and tumor progression. For example, Wu et al.^22^ found that miR-1273g-3p represses MAGEA3/6 expression in human CRC cells and tissues, which inhibits CRC tumorigenesis via activation of AMPK signaling. In this study, we plan to further identify the downstream targets of the LINC02418–miR-1273g-3p axis in CRC development and progression. We revealed that LINC02418 activated MELK as a ceRNA to absorb the MELK inhibitor miR-1273g-3p, which has not been reported. MELK is a member of the AMP-activated protein kinase-related kinase family, which controls a variety of biological processes, including cell cycle, cell proliferation, carcinogenesis, and apoptosis^25–27^. MELK has been shown to be differentially expressed in cancer stem cells or tumor-initiating cells, which drives cell cycle progression and tumor formation^28^. Furthermore, MELK is highly expressed in multiple human cancers, including prostate, gastric, and lung cancer, and is significantly associated with the poor overall survival of cancer patients^29–31^. Consistent with previous reports concerning MELK in CRC^32^, we found that MELK was upregulated in CRC compared with normal samples by analyzing the CRC mRNA microarray profile from the TCGA RNA sequencing data and the GEO datasets. The knockdown of MELK expression significantly reduced cell growth viability and colony formation viability. Moreover, rescue experiments determined that both the protein expression and tumor proliferative activity owing to MELK silencing could be partly reversed by LINC02418 overexpression, indicating that LINC02418 promoted CRC cell proliferation dependent on stimulating MELK expression.

The focus to fulfill the current clinical needs for the management of CRC patients has been on the search for new, noninvasive tools for an early and adequate diagnosis. Mounting evidence has suggested that cell-free lncRNAs could be detectable in the plasma and serum of cancer patients and therefore may be utilized as a tool for cancer diagnosis^33^. Recent studies have shown that lncRNAs could be enriched in exosomes and be secreted from the tumor cells to the body fluids via exosomes, and its expression profiling could reflect the evolution process of tumor cells in real time^34^. Because of the relatively stable characteristics of membrane structures, lncRNAs in exosomes have the strong advantages of being minimally invasive biomarkers for malignancy detection and prognosis^35^. An exosomal noncoding RNA-based diagnosis might provide a new strategy in clinical laboratory diagnostics since it has a remarkably high specificity and stability compared with other biomarker-based diagnoses^36^.Many studies have confirmed the opinions above. For example, tumor-originated exosomal lncUEGC1 showed the higher AUC values than CEA in discriminating gastric cancer from healthy individuals, and might be a potential circulating biomarker for early-stage gastric cancer^37^.

The detection of lncRNAs with the body fluid exosomes as carriers opens up a new perspective for the noninvasive “liquid biopsy” of tumors. Although numerous studies have focused on lncRNAs as potential tumor markers for cancer diagnosis and prognosis prediction, the diagnostic utility of serum exosomal lncRNAs in CRC has not yet been elucidated. In this study, our researches support the concept that lncRNAs in serum exosomes can exclude the influences of the external environment owing to their membrane-coated structure, providing more advantages than cell-free lncRNAs as tumor biomarkers. The ROC curves analyses showed that the AUC of serum exosomal LINC02418 was more higher than that of free circulating LINC02418, suggesting that the serum exosomal LINC02418 can be used as a biomarker for the diagnosis of CRC. The present study provided evidence that serum exosomal LINC02418 has potential diagnostic value for CRC, opening new opportunities for the clinical application of exosome research as novel, noninvasive tools.

In summary, this study provided evidence that the novel CRC-associated lncRNA LINC02418 was an oncogenic lncRNA that promoted CRC cell proliferation and inhibited cell apoptosis through the miR-1273g-3p-MELK axis. Moreover, we found that serum exosomal LINC02418 might serve as a novel diagnostic biomarker and a therapeutic target in CRC. However, serum exosomal LINC02418 detections have not been standardized, and the cutoff value of serum exosomal LINC02418 in this study may not be suitable for other studies. Therefore, additional large-scale studies, including larger clinical multicenter studies and functional analyses, are required to support the diagnostic performance of serum exosomal LINC02418 as a noninvasive biomarker in CRC.

## Materials and methods

### Human tissue and serum samples

We obtained 60 total paired CRC and adjacent non-tumor tissues from patients who were diagnosed with CRC based on histopathological diagnosis. All patients underwent surgical operation at the Second Hospital of Shandong University during January 2015 and January 2017. All enrolled patients did not receive preoperative chemotherapy or radiotherapy, and histopathological grades were staged according to the eighth TNM staging of the American Joint Committee on Cancer system classification. All collected tissue samples were immediately flash-frozen in liquid nitrogen and were stored at − 80 °C until use. The basic demography characteristics and clinical information of these 60 CRC patients were obtained from medical records (Table S3).

A total of 300 peripheral blood samples, including those from 155 patients with CRC and from 145 healthy individuals were collected from all participants before surgical resections or pharmacological interventions. First, the aberrant expression of LINC02418 in the serum was identified in 30 CRC patients and 20 healthy controls. Next, the diagnostic value of LINC02418 was validated in another independent cohort of 125 CRC patients and 125 healthy controls. The samples were collected at the Second Hospital of Shandong University between March 2016 and August 2018. Serum samples were isolated following a two-step centrifugation (1500 *g* for 10 min and 13800 *g* for 15 min at 4 °C) to eliminate cell sediments. All collected serum samples were transferred into 1.5 mL RNase- and DNase-free tubes and were stored at − 80 °C until use. Detailed clinical characteristics of enrolled CRC patients, including age, sex, tumor size, lymph node metastasis, and TNM stage, were listed in Table S4 and summarized in Table S5. The demographic information of the controls was also available in Table S5.

The study protocol was conducted in compliance with the ethical guidelines of the Declaration of Helsinki. Samples were collected under approval from the Research Ethics Committee of the Second Hospital of Shandong University, and informed written consent was obtained.

### Cell culture and transfection

Six human CRC cell lines (DLD-1, SW480, HT29, HCT116, SW1116, and LOVO), one normal colon epithelial cell line (FHC) and HEK293T were purchased from the Chinese Academy Medical Sciences (CAMS), China. FHC was cultured in Roswell Park Memorial Institute 1640 supplemented with 20% FBS (Australia Origin, Gibco, Carlsbad, CA, USA), and other tumor cell lines were cultured in DMEM supplemented with 10% FBS at 37 °C in an incubator with 5% CO_2_.

Tumor cells were transfected with siRNAs or plasmid vectors using Lipofectamine 2000 (Invitrogen, USA) according to the manufacturer’s instructions. The three individual LINC02418 siRNAs (si-LINC02418 1#, 2#, and 3#), three individual MELK siRNAs (si-MELK 1#, 2#, and 3#), a scrambled negative control siRNA, miR-1273g-3p mimics and inhibitors, miR-542-3p, miR-5186, miR-2277-3p, miR-3192-3p, miR-3193, and miR-4693-3p (GenePharma, Shanghai, China) used are listed in Table S6. PmirGLO-LINC02418-WT, pmirGLO-LINC02418-MUT, and pmirGLO empty vectors (Promega, Shanghai, China) were prepared using the Endo-Free Plasmid Mini Kit (Omega Bio-Tek, USA) and were transfected into tumor cells.

### Isolation and identification of exosomes from serum

To isolate exosomes from serum, 63 µL ExoQuick^TM^ solution (EXOQ5A-1, SBI System Biosciences, USA) was mixed well with 250 µL supernatant, followed by 30 min of incubation at 4 °C. Then, the exosomes were isolated by multistep centrifugation, including centrifugation at 4 °C at 1500 *g* for 30 min and at 1500 *g* for 5 min, to remove cells and debris. The sediments were resuspended in 50 µL PBS and were stored at − 80 °C until use.

The morphologies and ultrastructures of exosomes were analyzed using transmission electron microscopy. Exosome size distribution analyses were performed using the qNano® system (Izon Science, Oxford, UK) according to the manufacturer’s instructions. Exosome-specific proteins CD9 (CST, #13403) and TSG101 (Abcam, #ab125011) were determined using western blotting.

### Real-time qPCR analysis and primers

Total RNA was extracted from tissues, cultured cells and exosomes via TRIzol Reagent (Invitrogen, Carlsbad, CA, USA). RNA was reverse transcribed using random primers under the standard conditions for the PrimeScript RT reagent Kit (TaKara, Dalian, China). Real-time PCR analyses were performed with SYBR Premix Ex Taq (TaKara, Dalian China). RNA input was normalized to the level of glyceraldehyde-3-phosphate dehydrogenase (GAPDH). The specific primers used are as follows: LINC02418 forward, 5′-CCTTCCTTTCCAGCAGGACTT-3′ and reverse, 5′-GAGCAGAACCTGCCCAAAATG-3′; GAPDH forward, 5′-ACCCACTCCTCCACCTTTGAC-3′ and reverse, 5′-TGTTGCTGTAGCCAAATTCGTT-3′; and MELK forward, 5′-TCCTGTTGAGTGGCAAAGCA-3′ and reverse, 5′-CCTCCATTGTTTGCCTGTTGTT-3′. Primers for miR-542-3p, miR-5186, miR-2277-3p, miR-1273g-3p, miR-3192-3p, miR-3193, miR-4693-3p, and U6 were purchased from GeneCopoeia (Rockville, MD, USA). Data from qPCR analyses were analyzed by the △Ct method as described previously^18^.

### Western blotting and antibodies

Cells and exosomes were lysed with western/IP lysis buffer (Beyotime, Haimen, China), and tissues were lysed with radioimmunoprecipitation buffer extraction reagent (Solarbio, Beijing, China); both were supplied with a protease inhibitor cocktail (Roche Applied Science, Indianapolis, IN, USA). The whole-cell lysates were centrifuged at 12000 rpm for 15 min at 4 °C to extract proteins. Proteins were transferred to 0.22 mm polyvinylidene fluoride membranes (Merck-Millipore, Darmstadt, Germany) followed by separation with sodium dodecyl sulfate polyacrylamide gel electrophoresis, and the membranes were blocked with 5% nonfat milk and 1% Tween 20 in PBS for 1 h. Membranes were then incubated with primary antibodies at 4 °C overnight, which are listed as follows: anti-GAPDH (CST, #5174), anti-MELK (Abcam, #ab108529), anti-caspase substrates (CST, #8698), anti-cleaved caspase-3 (CST, #9664), anti-cleaved PARP (CST, #5625), anti-CD9 (CST, #13403), and anti-TSG101 (Abcam, #ab125011). The blots were then washed with PBS with 1% Tween 20 and were incubated with secondary antibodies conjugated to horseradish peroxidase (CST, #7074 or #7076). The signals were detected using HRP-based chemiluminescence analysis.

### Cell proliferation and colony formation assays

Cell growth and proliferation were monitored in real time using an xCELLigence RTCA DP instrument (ACEA Biosciences, San Diego, USA) for 72 h. For the colony formation assay, a total of 1000 SW1116 and HT29 cells were seeded into each well of a six-well plate and were maintained in DMEM containing 10% FBS for ~ 2 weeks. The medium was replaced every 3 days. Then, the colonies were fixed with methanol and were stained with 0.1% crystal violet (Solarbio, Beijing, China). The numbers of stained colonies were determined by counting.

### Flow cytometric analysis

Transfected cells were harvested after 48 h of transfection. For the cell apoptosis assay, after double staining with FITC-Annexin V and propidium iodide (PI), the cells were analyzed with flow cytometry (BD Biosciences) according to the manufacturer’s recommendations. The cells were classified as dead cells (Q1), early apoptotic cells (Q2), viable cells (Q3), and late apoptotic cells (Q4). For the cell cycle analysis, after staining with PI, the cells were analyzed by the Cycle TEST PLUS DNA Reagent Kit (BD Biosciences) following the protocol and were analyzed by FACScan. The percentages of the cells in the G0–G1, S, and G2–M phases were counted and compared.

### Dual-luciferase analysis

The complementary DNA fragments from the wild-type or mutant LINC02418 and MELK fragments were subcloned and inserted downstream of the luciferase gene within the pmirGLO-Basic luciferase reporter vector (Promega). Luciferase reporter vectors were stably co-transfected with scrambled negative control siRNAs as follows: miR-1273g-3p, miR-542-3p, miR-5186, miR-2277-3p, miR-3192-3p, miR-3193, and miR-4693-3p were co-transfected into SW1116 and HT29 cells. Luciferase activities were measured using a dual-luciferase reagent (Promega).

### Statistical analyses

Graph Pad Prism 6.0 and SPSS 13.0 software were used for the statistical analysis. The significant differences between the groups were assessed by Student’s *t* test or the Mann–Whitney *U* test as appropriate, and the Spearman rank-correlation analysis was used to calculate the correlation between LINC02418 and MELK. The tumor marker diagnostic performance was performed on MEDCALC 15.2.2 (Med-Calc, Mariakerke, Belgium). A *p* value < 0.05 was considered to be statistically significant. All results were expressed as the means ± standard deviation (SD) of three independent experiments.

## Supplementary information

Table S1

Table S2

Table S3

Table S4

Table S5

Table S6

Supplementary Figure S1.

Supplementary Figure S2.

Supplementary figure legends.
