# Supplementary material for: Long noncoding RNA LINC02418 regulates MELK expression by acting as a ceRNA and may serve as a diagnostic marker for colorectal cancer
Source: Cell Death Dis. 2019 Jul 29;10(8):568. doi: 10.1038/s41419-019-1804-x (PMC6662768; doi:10.1038/s41419-019-1804-x)
Supplement: Supplementary file 1 — Table S1 [file 41419_2019_1804_MOESM1_ESM.pdf]

Table S1. Correlation between concentrations of LINC02418 in tissues and clinic-pathological characteristics of 60 CRC patients [median (interquartile range)]

Parameters	Total case	LINC02418	*P* value
**Age (years)**			0.8967
≤62	30	1.72 (0.96-35.11)	
>62	30	5.27 (0.89-28.01)	
**Sex**			0.9285
Male	36	3.98 (1.01-28.70)	
Female	24	2.19 (0.73-27.71)	
**Lymph node metastasis**			0.5789
Negative	30	3.98 (1.09-15.17)	
Positive	30	1.86 (0.59-31.50)	
**Tumor size**			0.1858
≤5 cm	7	11.43 (1.80-49.18)	
>5 cm	53	1.89 (0.82-28.35)	
**Distant metastasis**			0.8946
No	49	2.75 (1.01-28.35)	
Yes	11	5.50 (0.64-71.01)	
**TNM stage**			0.8742
I	6	9.45 (1.55-22.85)	
II	19	2.75 (1.01-10.78)	
III	24	1.73 (0.55-32.89)	
IV	11	5.50 (0.64-71.01)	
