# Supplementary material for: Long noncoding RNA LINC02418 regulates MELK expression by acting as a ceRNA and may serve as a diagnostic marker for colorectal cancer
Source: Cell Death Dis. 2019 Jul 29;10(8):568. doi: 10.1038/s41419-019-1804-x (PMC6662768; doi:10.1038/s41419-019-1804-x)
Supplement: Supplementary file 2 — Table S2 [file 41419_2019_1804_MOESM2_ESM.pdf]

Table S2. Correlation between concentrations of exosomal lncRNAs and clinic-pathological characteristics of 125 CRC patients in validation set. [median (interquartile range)]

Parameters	Total case	LINC02418	*P* value
**Age(years)**			0.1639
<62	62	3.79 (2.06-8.98)	
≥62	63	4.77 (2.92-8.66)	
**Sex**			0.7732
Male	69	4.363 (2.338-7.211)	
Female	56	3.973 (2.18-58.60)	
**Tumor size**			0.8326
≤5 cm	21	4.36 (2.63-58.10)	
>5 cm	104	4.21 (2.18-8.30)	
**Lymph node metastasis**			0.9970
Negative	54	4.29 (2.67-7.23)	
Positive	71	4.19 (2.11-10.52)	
**Distant metastasis**			0.5627
No	114	4.29 (2.31-8.73)	
Yes	11	3.19 (1.69-94.69)	
**TNM stage**			0.9779
Tis	2	4.07 (3.77-4.36)	
I	16	3.75 (2.35-58.60)	
II	35	4.33 (2.94-6.94)	
III	61	4.49 (2.14-9.87)	
IV	11	3.19 (1.69-94.69)	
