# Supplementary material for: Long noncoding RNA LINC02418 regulates MELK expression by acting as a ceRNA and may serve as a diagnostic marker for colorectal cancer
Source: Cell Death Dis. 2019 Jul 29;10(8):568. doi: 10.1038/s41419-019-1804-x (PMC6662768; doi:10.1038/s41419-019-1804-x)
Supplement: Supplementary file 3 — Table S3 [file 41419_2019_1804_MOESM3_ESM.pdf]

Table S3. Demographic and clinical characteristics of individuals with CRC who provide tissues

Sample ID*	Gender	Age (Years)	T Stage	N Stage	M Stage	Tumor Location	Relative LINC02418 Expression**
SHSU-T-1	Female	62	T3	N0	M1	Rectum	1.144724161
SHSU-T-2	Male	60	T3	N2a	M1	Colon	6.147500725
SHSU-T-3	Male	62	T1	N0	M0	Colon	0.795536484
SHSU-T-4	Female	49	T3	N0	M0	Rectum	0.596667872
SHSU-T-5	Female	61	T2	N0	M0	Rectum	49.1800058
SHSU-T-6	Male	71	T3	N1b	M1	Colon	1.892115293
SHSU-T-7	Female	65	T3	N0	M0	Rectum	18.44298155
SHSU-T-8	Male	57	T3	N2b	M0	Rectum	1.017479692
SHSU-T-9	Male	61	T3	N0	M0	Rectum	132.9739652
SHSU-T-10	Female	48	T3	N0	M0	Rectum	1.484523571
SHSU-T-11	Male	58	T2	N0	M0	Rectum	1.802500925
SHSU-T-12	Male	64	T3	N2b	M0	Rectum	0.366021424
SHSU-T-13	Male	56	T3	N2b	M0	Rectum	1.32408891
SHSU-T-14	Female	62	T3	N1b	M0	Colon	1.630144665
SHSU-T-15	Female	39	T3	N2b	M1	Colorectum	0.064704058
SHSU-T-16	Male	77	T3	N2	M1	Rectum	5.502167273
SHSU-T-17	Female	24	T3	N1	M0	Rectum	0.602903914
SHSU-T-18	Male	60	T3	N2a	M0	Rectum	1.292352831
SHSU-T-19	Male	67	T3	N0	M0	Rectum	3.215403963
SHSU-T-20	Male	62	T1	N0	M0	Colon	14.07438521
SHSU-T-21	Male	73	T3	N1b	M0	Colon	164.2785149
SHSU-T-22	Female	69	T3	N0	M1	Colon	8.78474251
SHSU-T-23	Male	79	T3	N0	M1	Colon	0.147624083
SHSU-T-24	Male	53	T3	N0	M0	Rectum	1.479387509

SHSU-T-25	Male	75	T3	N1a	M0	Rectum	0.535886731
SHSU-T-26	Female	72	T3	N0	M0	Colon	66.94943615
SHSU-T-27	Female	77	T3	N1b	M0	Rectum	30.80302218
SHSU-T-28	Male	35	T3	N1b	M0	Rectum	0.131670129
SHSU-T-29	Female	68	T3	N2a	M0	Colon	0.272626933
SHSU-T-30	Male	62	T3	N0	M0	Colon	6.868523492
SHSU-T-31	Male	55	T3	N2b	M0	Colon	0.453759578
SHSU-T-32	Male	64	T3	N1b	M0	Colon	38.05462768
SHSU-T-33	Female	63	T3	N2a	M0	Rectum	0.346277367
SHSU-T-34	Male	73	T3	N0	M0	Rectum	0.299369676
SHSU-T-35	Female	61	T3	N2b	M1	Colorectum	0.639492791
SHSU-T-36	Male	73	T2	N0	M0	Rectum	11.43195312
SHSU-T-37	Male	51	T3	N0	M0	Rectum	390.7223575
SHSU-T-38	Male	62	T3	N1a	M0	Colon	1.8276629
SHSU-T-39	Female	66	T3	N2b	M1	Colon	232.3249038
SHSU-T-40	Male	42	T3	N2a	M0	Colon	39.6706464
SHSU-T-41	Male	73	T3	N2a	M0	Colon	27.6651914
SHSU-T-42	Female	76	T3	N0	M0	Colon	2.751083636
SHSU-T-43	Male	59	T2	N1	M0	Rectum	263.1971396
SHSU-T-44	Male	44	T3	N0	M0	Rectum	4.740371084
SHSU-T-45	Male	81	T3	N2a	M0	Colon	6.190259974
SHSU-T-46	Female	75	T2	N0	M0	Rectum	7.464263932
SHSU-T-47	Male	60	T3	N0	M0	Colon	8.456144324
SHSU-T-48	Male	68	T3	N0	M0	Colon	0.378929142
SHSU-T-49	Female	88	T3	N0	M0	Rectum	1.00695555
SHSU-T-50	Female	81	T3	N0	M0	Rectum	1.117287138
SHSU-T-51	Female	65	T3	N2a	M0	Rectum	533.7424696
SHSU-T-52	Female	78	T3	N0	M0	Rectum	10.77786861
SHSU-T-53	Male	74	T3	N1b	M0	Colon	29.04061297

SHSU-T-54	Female	75	T3	N2b	M0	Colon	5.028053498
SHSU-T-55	Female	66	T3	N0	M0	Rectum	1.526259209
SHSU-T-56	Male	37	T3	N2b	M0	Rectum	1.366040257
SHSU-T-57	Male	66	T3	N0	M0	Colon	1.003471749
SHSU-T-58	Female	61	T3	N0	M1	Rectum	596.3435963
SHSU-T-59	Male	51	T3	N2a	M0	Rectum	33.59093388
SHSU-T-60	Male	32	T3	N0	M1	Colorectum	71.01244621

* SHSU-T, paired cancer and normal tissues were from 60 CRC patients who underwent surgical operation at the Second Hospital of Shandong University (SHSU).

** Relative LINC02418 Expression, relative expression of LINC02418 compared to matched adjacent normal tissues by qPCR.
