# Supplementary material for: Long noncoding RNA LINC02418 regulates MELK expression by acting as a ceRNA and may serve as a diagnostic marker for colorectal cancer
Source: Cell Death Dis. 2019 Jul 29;10(8):568. doi: 10.1038/s41419-019-1804-x (PMC6662768; doi:10.1038/s41419-019-1804-x)
Supplement: Supplementary file 4 — Table S4 [file 41419_2019_1804_MOESM4_ESM.pdf]

Table S4. Demographic and clinical characteristics of individuals with CRC who provide serums

Sample ID*	Gender	Age (Years)	T Stage	N Stage	M Stage	Tumor Location
SHSU-S-1	Male	84	T3	N2b	M0	Rectum
SHSU-S-2	Male	57	T3	N1b	M0	Rectum
SHSU-S-3	Female	70	T3	N0	M0	Rectum
SHSU-S-4	Male	63	T3	N1b	M0	Colorectum
SHSU-S-5	Male	55	T3	N0	M0	Colon
SHSU-S-6	Male	84	T3	N0	M0	Colon
SHSU-S-7	Female	47	T3	N0	M0	Colorectum
SHSU-S-8	Female	58	T2	N0	M0	Rectum
SHSU-S-9	Male	78	T3	N0	M0	Colon
SHSU-S-10	Male	55	T3	N1b	M0	Rectum
SHSU-S-11	Female	84	T4a	N0	M0	Colon
SHSU-S-12	Female	42	T2	N1b	M0	Colon
SHSU-S-13	Male	70	T3	N2a	M0	Rectum
SHSU-S-14	Female	45	T3	N1b	M0	Rectum
SHSU-S-15	Male	70	T3	N0	M0	Rectum
SHSU-S-16	Male	50	T3	N1b	M0	Colon
SHSU-S-17	Female	70	Tis	N0	M0	Colon
SHSU-S-18	Male	41	T3	N2b	M0	Rectum
SHSU-S-19	Female	66	T3	N1	M0	Colon
SHSU-S-20	Male	80	T3	N1b	M0	Colon
SHSU-S-21	Female	55	T3	N2a	M0	Rectum
SHSU-S-22	Female	41	T3	N0	M0	Rectum
SHSU-S-23	Male	68	T0	N0	M0	Rectum
SHSU-S-24	Female	53	T3	N1b	M0	Rectum
SHSU-S-25	Female	62	T3	N0	M0	Colon
SHSU-S-26	Male	69	T2	N0	M0	Rectum
SHSU-S-27	Male	78	T3	N2a	M0	Colon

SHSU-S-28	Male	41	T3	N2a	M0	Colon
SHSU-S-29	Male	75	T1	N0	M0	Rectum
SHSU-S-30	Male	35	T3	N0	M0	Colon
SHSU-S-31	Female	41	T3	N1b	M0	Rectum
SHSU-S-32	Female	45	T2	N0	M0	Rectum
SHSU-S-33	Female	54	T2	N0	M0	Colon
SHSU-S-34	Female	61	T4a	N1b	M0	Rectum
SHSU-S-35	Male	68	T3	N0	M0	Rectum
SHSU-S-36	Female	54	T2	N0	M0	Rectum
SHSU-S-37	Female	41	T3	N0	M0	Rectum
SHSU-S-38	Male	59	T3	N1a	M0	Colon
SHSU-S-39	Male	82	T4a	N1a	M0	Rectum
SHSU-S-40	Male	75	T3	N0	M0	Rectum
SHSU-S-41	Male	55	T3	N2a	M0	Rectum
SHSU-S-42	Female	66	T2	N0	M0	Colon
SHSU-S-43	Female	73	T3	N1b	M1	Colon
SHSU-S-44	Male	61	T3	N1a	M0	Colon
SHSU-S-45	Female	61	T3	N1a	M0	Colon
SHSU-S-46	Male	62	T3	N1a	M0	Rectum
SHSU-S-47	Female	36	T3	N2a	M0	Colon
SHSU-S-48	Male	68	T3	N2a	M1	Colon
SHSU-S-49	Female	61	T3	N0	M0	Rectum
SHSU-S-50	Female	60	T3	N1b	M0	Rectum
SHSU-S-51	Male	51	T1	N0	M0	Colon
SHSU-S-52	Male	63	T2	N1a	M0	Colon
SHSU-S-53	Male	62	T3	N0	M1	Rectum
SHSU-S-54	Male	48	T3	N0	M0	Colon
SHSU-S-55	Female	84	T3	N2a	M0	Rectum
SHSU-S-56	Female	62	T2	N0	M0	Rectum
SHSU-S-57	Male	66	T3	N2a	M0	Rectum
SHSU-S-58	Female	58	T2	N0	M0	Colon
SHSU-S-59	Male	60	T4a	N1a	M0	Rectum
SHSU-S-60	Male	62	T3	N2a	M0	Rectum

SHSU-S-61	Female	88	T3	N1a	M0	Colon
SHSU-S-62	Male	66	T4a	N1a	M0	Rectum
SHSU-S-63	Male	52	T3	N0	M0	Rectum
SHSU-S-64	Male	34	T4a	N0	M0	Rectum
SHSU-S-65	Female	70	T4a	N0	M0	Colon
SHSU-S-66	Female	73	T4a	N1a	M0	Rectum
SHSU-S-67	Male	24	T3	N0	M0	Rectum
SHSU-S-68	Female	54	T3	N1a	M0	Rectum
SHSU-S-69	Male	59	T4a	N2a	M0	Rectum
SHSU-S-70	Female	76	T2	N0	M0	Colon
SHSU-S-71	Female	60	T3	N1a	M0	Colon
SHSU-S-72	Female	54	T4a	N1a	M0	Colon
SHSU-S-73	Male	65	T3	N0	M0	Colon
SHSU-S-74	Male	80	T3	N0	M0	Rectum
SHSU-S-75	Male	59	T3	N2a	M0	Rectum
SHSU-S-76	Female	66	T3	N2a	M0	Colon
SHSU-S-77	Male	53	T4a	N0	M0	Rectum
SHSU-S-78	Male	28	T3	N2b	M0	Rectum
SHSU-S-79	Male	41	T3	N0	M0	Colon
SHSU-S-80	Male	67	T3	N2b	M0	Rectum
SHSU-S-81	Male	49	T3	N0	M0	Rectum
SHSU-S-82	Female	59	T4a	N2b	M0	Rectum
SHSU-S-83	Male	52	T2	N1a	M0	Rectum
SHSU-S-84	Male	45	T4a	N1a	M0	Rectum
SHSU-S-85	Female	78	T4a	N0	M0	Colon
SHSU-S-86	Male	63	T4a	N0	M0	Colon
SHSU-S-87	Female	58	T4a	N0	M0	Rectum
SHSU-S-88	Male	46	T3	N0	M0	Rectum
SHSU-S-89	Male	69	T3	N1a	M0	Colon
SHSU-S-90	Male	78	T4a	N1a	M0	Colon
SHSU-S-91	Female	71	T4a	N0	M0	Rectum
SHSU-S-92	Male	77	T3	N2b	M1	Rectum
SHSU-S-93	Female	69	T3	N1a	M1	Rectum

SHSU-S-94	Male	54	T4a	N1a	M0	Colon
SHSU-S-95	Female	69	T3	N1a	M0	Colon
SHSU-S-96	Male	70	T4a	N0	M0	Rectum
SHSU-S-97	Female	74	T3	N2b	M0	Colon
SHSU-S-98	Female	68	T3	N0	M0	Rectum
SHSU-S-99	Male	48	T4a	N0	M0	Rectum
SHSU-S-100	Male	56	Tis	N0	M0	Rectum
SHSU-S-101	Male	70	T3	N0	M0	Rectum
SHSU-S-102	Male	66	T4a	N0	M0	Rectum
SHSU-S-103	Female	76	T3	N0	M0	Colon
SHSU-S-104	Female	60	T4a	N1a	M0	Rectum
SHSU-S-105	Male	63	T3	N1a	M0	Rectum
SHSU-S-106	Male	60	T4a	N0	M0	Rectum
SHSU-S-107	Male	58	T3	N1a	M0	Colorectum
SHSU-S-108	Male	59	T3	N0	M0	Rectum
SHSU-S-109	Male	66	T4a	N0	M0	Colorectum
SHSU-S-110	Male	68	T4a	N0	M0	Rectum
SHSU-S-111	Male	54	T3	N1a	M0	Rectum
SHSU-S-112	Male	73	T3	N0	M0	Colon
SHSU-S-113	Female	61	T2	N0	M0	Rectum
SHSU-S-114	Female	45	T3	N0	M0	Rectum
SHSU-S-115	Male	25	T4a	N1a	M0	Rectum
SHSU-S-116	Male	72	T4a	N1a	M0	Rectum
SHSU-S-117	Male	45	T3	N0	M0	Rectum
SHSU-S-118	Male	48	T4a	N1a	M0	Rectum
SHSU-S-119	Female	71	T4a	N1a	M0	Rectum
SHSU-S-120	Female	53	T2	N0	M0	Colon
SHSU-S-121	Female	78	T4a	N2b	M0	Rectum
SHSU-S-122	Female	60	T2	N0	M0	Rectum
SHSU-S-123	Male	67	T2	N0	M0	Rectum
SHSU-S-124	Female	73	T4a	N0	M0	Colorectum
SHSU-S-125	Female	69	T1	N0	M0	Colon
SHSU-S-126	Male	70	T3	N2a	M0	Colon

SHSU-S-127	Male	66	T2	N0	M0	Colon
SHSU-S-128	Female	73	T4a	N0	M0	Rectum
SHSU-S-129	Female	81	T3	N2a	M0	Rectum
SHSU-S-130	Female	54	T3	N1a	M0	Rectum
SHSU-S-131	Female	50	T2	N1a	M0	Colon
SHSU-S-132	Female	78	T3	N2a	M0	Colon
SHSU-S-133	Male	50	T2	N0	M0	Colon
SHSU-S-134	Male	66	T4a	N2b	M0	Colon
SHSU-S-135	Male	61	T3	N2a	M0	Rectum
SHSU-S-136	Female	65	T3	N2b	M1	Colon
SHSU-S-137	Female	49	T4a	N1a	M0	Colorectum
SHSU-S-138	Female	71	T3	N1a	M0	Colon
SHSU-S-139	Male	68	T3	N0	M0	Colon
SHSU-S-140	Female	77	Tis	N0	M0	Rectum
SHSU-S-141	Female	61	T3	N1a	M0	Rectum
SHSU-S-142	Male	56	T4a	N2b	M1	Colorectum
SHSU-S-143	Female	34	T4a	N2b	M1	Colorectum
SHSU-S-144	Female	59	T3	N1a	M0	Colon
SHSU-S-145	Female	69	T3	N2b	M1	Rectum
SHSU-S-146	Male	76	T3	N1a	M0	Rectum
SHSU-S-147	Male	66	T2	N0	M0	Rectum
SHSU-S-148	Male	52	T4a	N2b	M0	Rectum
SHSU-S-149	Male	78	T3	N1a	M0	Rectum
SHSU-S-150	Male	46	T4a	N2b	M1	Colon
SHSU-S-151	Female	48	T3	N2b	M0	Colon
SHSU-S-152	Female	83	T3	N2a	M0	Colorectum
SHSU-S-153	Male	84	T3	N2b	M0	Colon
SHSU-S-154	Male	57	T3	N1b	M0	Rectum
SHSU-S-155	Male	70	T3	N1a	M1	Rectum

* SHSU-S, serums were from 155 CRC patients at the Second Hospital of Shandong University (SHSU). SHSU-S-1 - SHSU-S-155 were used to extract cell-free RNA from

serums, and SHSU-S-31 - SHSU-S-155 were used to isolate exosomes from serums.
