# Supplementary material for: Long noncoding RNA LINC02418 regulates MELK expression by acting as a ceRNA and may serve as a diagnostic marker for colorectal cancer
Source: Cell Death Dis. 2019 Jul 29;10(8):568. doi: 10.1038/s41419-019-1804-x (PMC6662768; doi:10.1038/s41419-019-1804-x)
Supplement: Supplementary file 5 — Table S5 [file 41419_2019_1804_MOESM5_ESM.pdf]

Table S5. Clinic-pathological characteristics of patients and demographic information of controls enrolled in the study.

Variable	CRC patients n=155	Healthy individuals n=145
**Age (years)**		
< 62	76	67
≥62	79	78
**Sex**		
Male	87	84
Female	68	61
**Tumor size**		
≤5 cm	27	NA
>5 cm	128	NA
**Lymph node metastasis**		
Negative	69	NA
Positive	86	NA
**Distant metastasis**		
No	144	NA
Yes	11	NA
**TNM stage**		
Tis	4	NA
I	19	NA
II	45	NA
III	76	NA
IV	11	NA

NA, not available.
