# Supplementary material for: Long noncoding RNA LINC02418 regulates MELK expression by acting as a ceRNA and may serve as a diagnostic marker for colorectal cancer
Source: Cell Death Dis. 2019 Jul 29;10(8):568. doi: 10.1038/s41419-019-1804-x (PMC6662768; doi:10.1038/s41419-019-1804-x)
Supplement: Supplementary file 6 — Table S6 [file 41419_2019_1804_MOESM6_ESM.pdf]

Table S6. SiRNA sequences are as following

LINC02418-si1#	CCACUGGAAUUAUAGCUAATT
LINC02418-si2#	CCGCAUGACCCAUCUGAUUTT
LINC02418-si3#	CCAGAAGAGUAACCACCAATT
MELK-si#1	GGCGGGAUUAAUAGACUAUTT
MELK -si2#	GCCAAAGACUCCAGUUAAUTT
MELK -si3#	CCUGGAUCAUGCAAGAUUATT
negative control siRNA	UUCUCCGAACGUGUCACGUTT
miR-1273g-3p mimics	ACCACUGCACUCCAGCCUGAG
miR-1273g-3p inhibitor	CUCAGGCUGGAGUGCAGUGGU
miR-542-3p mimics	UGUGACAGAUUGAUAACUGAAA
miR-5186 mimics	AGAGAUUGGUAGAAAUCAGGU
miR-2277-3p mimics	UGACAGCGCCCUGCCUGGCUC
miR-3192-3p mimics	CUCUGAUCGCCCUCUCAGCUC
miR-3193 mimics	UCCUGCGUAGGAUCUGAGGAGU
miR-4693-3p mimics	UGAGAGUGGAAUUCACAGUAUUU
