# Supplementary figures and images for: Long noncoding RNA LINC02418 regulates MELK expression by acting as a ceRNA and may serve as a diagnostic marker for colorectal cancer
Source: Cell Death Dis. 2019 Jul 29;10(8):568. doi: 10.1038/s41419-019-1804-x (PMC6662768; doi:10.1038/s41419-019-1804-x)

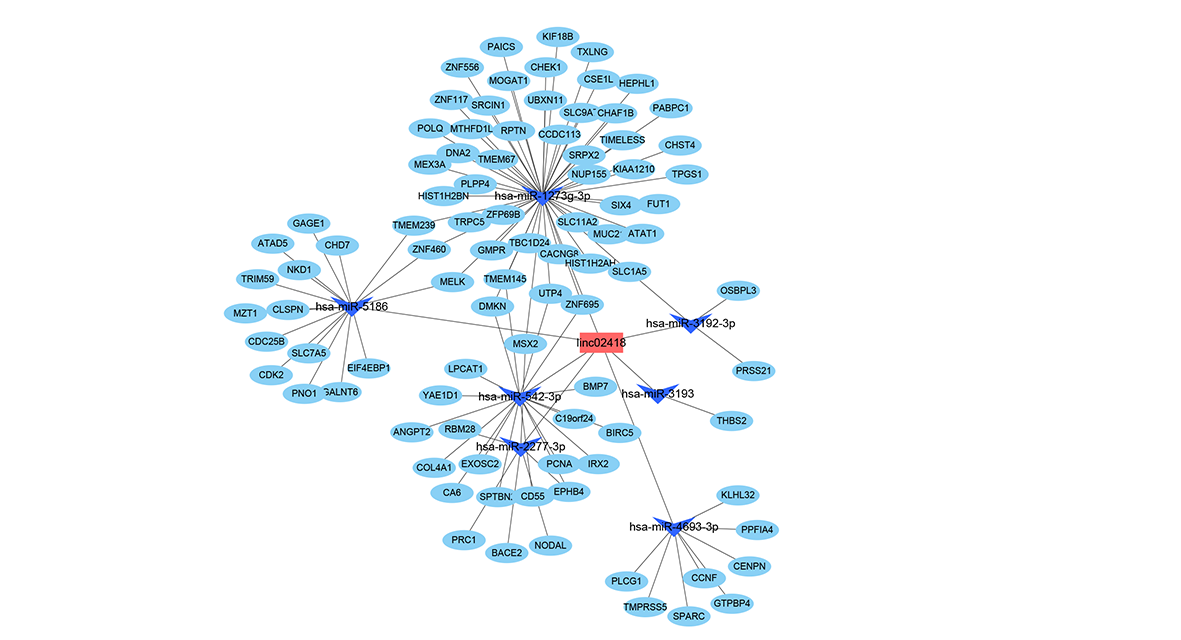

Supplement: Supplementary file 7 — Supplementary Figure S1. [file 41419_2019_1804_MOESM7_ESM.tif]

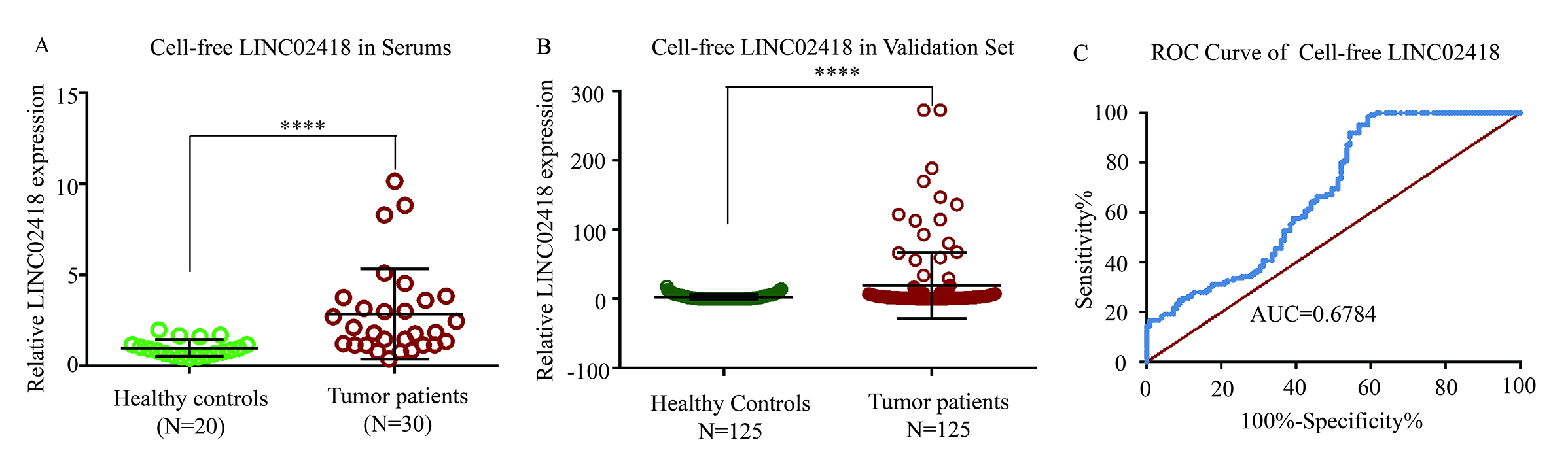

Supplement: Supplementary file 8 — Supplementary Figure S2. [file 41419_2019_1804_MOESM8_ESM.tif]
