# Supplementary material for: Long noncoding RNA LINC02418 regulates MELK expression by acting as a ceRNA and may serve as a diagnostic marker for colorectal cancer
Source: Cell Death Dis. 2019 Jul 29;10(8):568. doi: 10.1038/s41419-019-1804-x (PMC6662768; doi:10.1038/s41419-019-1804-x)
Supplement: Supplementary file 9 — Supplementary figure legends. [file 41419_2019_1804_MOESM9_ESM.doc]

**Supplementary Fig. S1 LINC02418-miRNA-mRNA network.**

**Supplementary Fig. S2 Verification of expression of the LINC02418 in serum. a** RNA expression of cell-free LINC02418 was measured in the serum of healthy controls (n = 20) and the serum of CRC patients (n = 30) using qPCR. **b** RNA expression of cell-free LINC02418 was measured in validation set (125 CRC patients and 125 healthy controls). **c** ROC curve for serum cell-free LINC02418 for the discrimination of patients with CRC from normal healthy individuals. ****p < 0.0001.
